# Theta and gamma oscillations in the rat hippocampus support the discrimination of object displacement in a recognition memory task

**DOI:** 10.3389/fnbeh.2022.970083

**Published:** 2022-12-21

**Authors:** Lívia Neves, Bruno Lobão-Soares, Ana Paula de Castro Araujo, Alan Michel Bezerra Furtunato, Izabela Paiva, Nicholy Souza, Anne Kelly Morais, George Nascimento, Elaine Gavioli, Adriano Bretanha Lopes Tort, Flávio Freitas Barbosa, Hindiael Belchior

**Affiliations:** ^1^Graduate Program in Psychobiology, Federal University of Rio Grande do Norte, Natal, RN, Brazil; ^2^Department of Biophysics and Pharmacology, Federal University of Rio Grande do Norte, Natal, RN, Brazil; ^3^Graduate Program in Cognitive Neuroscience and Behavior, Federal University of Paraíba, João Pessoa, PB, Brazil; ^4^Department of Psychology, Federal University of Paraíba, João Pessoa, PB, Brazil; ^5^Brain Institute, Federal University of Rio Grande do Norte, Natal, RN, Brazil; ^6^Department of Biomedical Engineering, Federal University of Rio Grande do Norte, Natal, RN, Brazil; ^7^Department of Physical Education, Federal University of Rio Grande do Norte, Natal, RN, Brazil

**Keywords:** hippocampus, local field potentials, recognition memory, spatial displacement of objects, pattern separation

## Abstract

Episodic memory depends on the recollection of spatial and temporal aspects of past experiences in which the hippocampus plays a critical role. Studies on hippocampal lesions in rodents have shown that dentate gyrus (DG) and CA3 are necessary to detect object displacement in memory tasks. However, the understanding of real-time oscillatory activity underlying memory discrimination of subtle and pronounced displacements remains elusive. Here, we chronically implanted microelectrode arrays in adult male Wistar rats to record network oscillations from DG, CA3, and CA1 of the dorsal hippocampus while animals executed an object recognition task of high and low spatial displacement tests (HD: 108 cm, and LD: 54 cm, respectively). Behavioral analysis showed that the animals discriminate between stationary and displaced objects in the HD but not LD conditions. To investigate the hypothesis that theta and gamma oscillations in different areas of the hippocampus support discrimination processes in a recognition memory task, we compared epochs of object exploration between HD and LD conditions as well as displaced and stationary objects. We observed that object exploration epochs were accompanied by strong rhythmic activity in the theta frequency (6–12 Hz) band in the three hippocampal areas. Comparison between test conditions revealed higher theta band power and higher theta-gamma phase-amplitude coupling in the DG during HD than LD conditions. Similarly, direct comparison between displaced and stationary objects within the HD test showed higher theta band power in CA3 during exploration of displaced objects. Moreover, the discrimination index between displaced and stationary objects directly correlated with CA1 gamma band power in epochs of object exploration. We thus conclude that theta and gamma oscillations in the dorsal hippocampus support the successful discrimination of object displacement in a recognition memory task.

## Introduction

Living in a complex and dynamic world must require flexible memory systems capable of detecting subtle changes in the environment. The spatial and temporal aspects of past experiences are fundamental components in the recollection of episodic memories (Tulving, 2002; Eichenbaum et al., 2012). Once retrieved, previously acquired information is compared with current sensory inputs allowing the detection of contextual changes, which is critical to distinguish among similar episodic memories. This mnemonic process, named pattern separation, implements fine distinctions between similar patterns.

Researchers have been using spontaneous object exploration tasks as a tool to assess pattern separation and recognition memory in rodents, which exhibit a natural drive to detect and explore novelty in their environment (Barbosa and Silva, 2018; Wang et al., 2021). For instance, rats spend more time exploring a new object when compared to a familiar one in the novel object recognition task (Chao et al., 2020). A variant of this task, called novel object location task (NOL), evaluates spatial memory performance in rats. The animals are allowed to explore two equal objects in a familiar arena during the sample phase, and after a given interval one of these objects is moved to a new location. It is expected that rats spend more time exploring the displaced object relative to the stationary one (Ennaceur, 2010; Cohen and Stackman, 2015; Araujo et al., 2021). Hunsaker and Kesner (2008) have developed a NOL paradigm that sets different levels of object displacement as a tool to study spatial pattern separation. High displacements (HD) are expected to be more easily detectable by rats when compared to low displacement (LD) due to spatial interference between close object positions. The authors showed that hippocampal lesions in the dentate gyrus (DG) and CA3 impaired the discrimination of displaced objects. Specifically, DG lesions disrupted fine spatial discrimination and CA3 lesions affected global detection of alterations in the environment (Hunsaker and Kesner, 2008).

In parallel to lesion and behavioral studies, electrophysiological recordings have also implicated the hippocampus in recognition memory processes (Kemp and Manahan-Vaughan, 2004; França et al., 2015). Trimper et al. (2017) reported a critical role of the dorsal DG and CA3 slow gamma oscillations (30–60 Hz) during retrieval in an object recognition task. In particular, they have found the highest slow gamma power when rats explored novel objects, followed by familiar objects in swapped positions. The lowest level of slow gamma power occurred when rats explored familiar objects at the same locations, i.e., control groups. These results indicate a role for the DG and CA3 slow gamma activity in associative recognition memory for objects and their locations. Recently, Wang et al. (2021) detected an increase in ventral CA1 theta band power and in hippocampus-prefrontal theta band synchrony during the exploration of novel in opposition to familiar objects in a novel object recognition memory test. Additionally, a disturbed hippocampal-prefrontal connectivity performed by optogenetic silencing resulted in reduced theta synchrony and impaired novel object recognition. In contrast, Zheng et al. (2016) have found that fast gamma (60–100 Hz) band power in the dorsal CA1 was stronger during the retrieval as opposed to the sample phase when tested on a novel object in a novel location recognition task. Taken together, these studies reveal that hippocampal oscillations mediate mnemonic information during object recognition tasks, such as those involving changes in identity and positioning of objects.

Despite evidence pointing to the involvement of hippocampal theta and gamma rhythms in the discrimination of spatial object displacements, the role of different hippocampal areas remains elusive. Thus, here we postulate that theta and gamma oscillations in specific areas of the hippocampus support recognition memory while rats explore stationary and displaced objects. To test this hypothesis, we chronically implanted microelectrode arrays to simultaneously record from DG, CA3, and CA1 areas of the dorsal hippocampus of rats submitted to an object recognition task of high and low spatial displacements.

## Materials and methods

### Animals

We used eight male *Wistar* rats (3 months old; ∼350 g) provided by the Biosciences Center Central Bioterium of the Federal University of Rio Grande do Norte. They were housed in a maximum number of four in standard cages (30 × 20 × 19 cm) on a 12 h/12 h light-dark cycle (lights on at 6 am) with food and water *ad libitum*. All the experiments were conducted on the light phase of the cycle. Experiments were approved by the Ethics Committee on the Use of Animals (CEUA/UFRN, permit n° 52/2016) and in accordance with the Guide for the Care and Use of Laboratory Animals, 8th Edition (National Research Council [NRC], 2011).

### Electrodes

**FIGURE 1:**
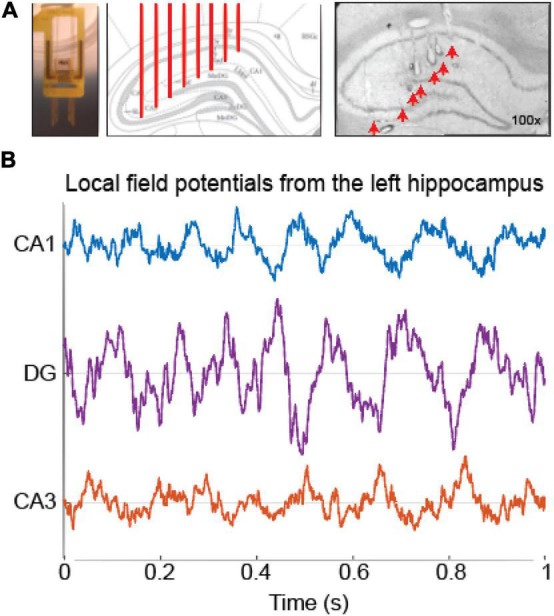
Histology and electrophysiological recordings. **(A)** Representative picture of: a 16-ch microelectrode array designed to bilaterally record from DG, CA3, and CA1 areas of the dorsal hippocampus (left); the targeted anatomical areas (3,6 mm posterior to Bregma; Paxinos and Watson, 2013) and electrode positions in the hippocampus (red vertical lines, middle); a representative 50-μm Nissl-stained coronal section of the left hemisphere of the dorsal hippocampus (right). Red arrows point to tissue lesions caused by electric currents individually applied at each electrode. **(B)** Representative examples of simultaneous recordings of hippocampal local field potentials. Traces on different colors show selected electrodes positioned in each hippocampal area: CA1 (blue), DG (purple), and CA3 (red). Note the phase reversal of the theta cycle between the DG and CA1/CA3.

We built microelectrode arrays (16 electrodes, 50 μm diameter Teflon-coated tungsten wires, California Fine Wire) designed to target the dorsal hippocampus of both hemispheres [−3.6 mm AP, ± 3.0 mm ML, according to Paxinos and Watson (2013)]. Electrodes were arranged in two 1 × 8 bundles with an inter-electrode lateral spacing of 250 μm, and an inter-electrode depth difference of 200 μm creating a stair design (Figure 1A). The eight microelectrodes were distributed across the laminar profile of the hippocampus (from 2.1 to 3.6 mm DV) to record electrophysiological signals from CA1, CA3, and DG from both hemispheres. The electrode impedance was reduced to ∼0.5 MOhms at 1 kHz in a gold solution with carbon nanotubes using NanoZ (Neuralynx) previously to surgery in accordance with previous studies (Ferguson et al., 2009).

### Surgery

Animals were treated with atropine (0.04 mg/kg, s.c.), anesthetized with ketamine and xylazine (respectively, 100 mg/kg and 8 mg/kg, i.p.), and placed in a stereotaxic (Insight Equipamentos). Rectangular craniotomies were made to allow electrode insertion into the brain tissue. Two stainless steel screws soldered to a silver wire were implanted in the occipital cranial bone to provide ground and reference. Four additional stainless steel screws were positioned into the parietal and frontal cranial bones to provide mechanical support to the electrode arrays. Acrylic resin was used to cement the electrode array at the final target position. After surgery, animals were treated with anti-inflammatory (flunixin-meglumine at 2.5 mg/kg, i.p.), anti-biotic (enrofloxacin at 10 mg/kg, s.c.), and analgesic (paracetamol at 200 mg/kg, oral) for the following 3 days.

### Experimental protocol

After recovery, animals were allowed to a daily session of 30 min of acclimation in the experimental room before starting any procedure. During five consecutive days, rats were handled for 20 min in order to reduce stress related to the presence and physical contact with the experimenters. In the first two days, handling was performed in the homecage collectively, and in the following 3 days rats were handled individually. Additional handling sessions of 5 min were performed on task days. In the next 4 days, animals were habituated to the apparatus (the rest box used to hold animals during the inter-task intervals and the open field, see description below) for 10 min per day. No object was presented in the open field in the habituation sessions. See Supplementary Figure 1 for a detailed schema of the experimental design.

The object recognition task was performed in an all-black circular open field (height 45 cm, diameter 118 cm) with four proximal cues on the arena walls and another four additional distal cues on the walls of the experimental room. We used two copies of the same object made of glass or ceramic materials (see pictures of the objects in Supplementary Figure 2). Objects were positioned radially in the open field 4 cm distant from the walls. At trial start, animals were placed in the center of the open field facing the northern direction. During the 10-min intervals of the task, rats were placed on a rest box (height 45 cm, width 45 cm, length 45 cm) of white walls and a black floor.

### Novel object location recognition task

**FIGURE 2:**
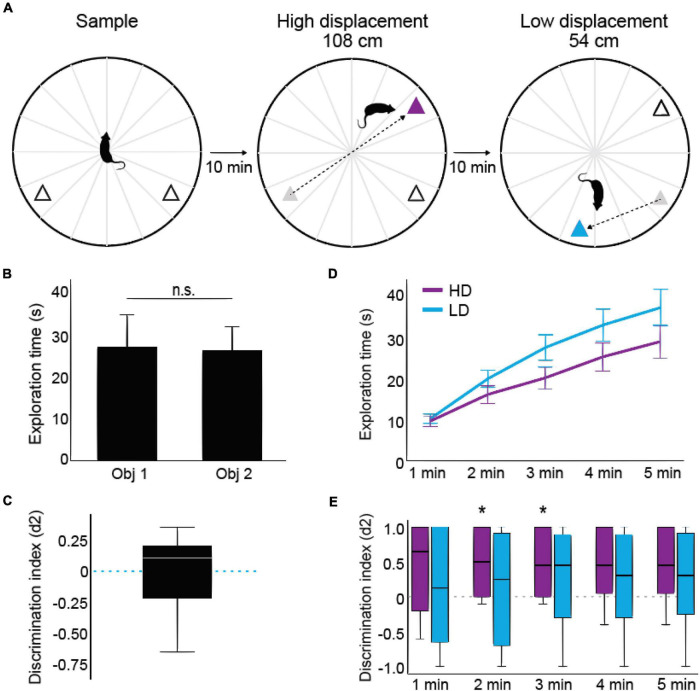
Experimental design and behavioral performance. **(A)** Schematic illustration of the object recognition task with high and low spatial displacement tests. In the sample phase, rats explored two identical objects (triangles) in a circular open field (118 cm diameter) for 5 min. In the high and low displacement tests, one object was spatially displaced by 108 cm and another by 54 cm, respectively. **(B)** Total exploration time of each object during the sample session. Bars represent means and error bars represent SEM. **(C)** Discrimination index among objects [(tDO – tSO)/(tDO + tSO)] calculated during the sample session. The whisker plot shows the distribution of discrimination index values, where the white line depicts median, black bars represent upper and lower quartiles, and error bars represent values outside the middle 50%. Dashed line depicts chance values (i.e., rats devoted the same time to exploring both objects). **(D)** Minute-by-minute cumulative analysis of the time of object exploration during the high and low spatial displacement tests (purple and cyan, respectively). Lines depict means and error bars represent SEM. **(E)** Minute-by-minute cumulative analysis of the discrimination index in HD and LD tests (purple and cyan, respectively). Black lines depict medians, bars represent upper and lower quartiles, and error bars represent values outside the middle 50%. Asterisks indicate *p* < 0.05 against zero (i.e., chance levels; Wilcoxon signed-rank test), *n* = 8 animals.

To assess the behavioral correlates of spatial novelty detection we adapted a protocol from Hunsaker and Kesner (2008). The protocol consisted of one 5-min sample trial in which rats were presented to two identical objects and two 5-min test trials in which one of the objects was moved to a new place (Figure 2A). Spatial displacements were performed at large or at small distances. When the object was moved 108 cm from its previous position, the test was defined as a high spatial displacement (HD, as shown in Figure 2A). When the object was moved 54 cm from its previous position, the test was defined as a low spatial displacement (LD). Half of the animals were first exposed to HD followed by LD, while the other half had the exposure given in the opposite order. The spatial positions and type (ceramic or glass) of objects were randomized among animals.

### Data collection

Continuous electrophysiological recordings were performed using a headstage preamplifier wired-coupled to a multi-channel recording system (RHA2116, Intan Technologies). Raw electrophysiological signals were filtered between 0.02 and 20 kHz and recorded at 30 kHz. Animal behavior was recorded in video by a high-definition digital camera positioned above the rest box and the open field apparatus (1080 × 720 pixels at 30 frames/s, Logitech C920). Video and electrophysiological recordings were synchronized by a microcontroller (Arduino Uno) and stored for posterior analysis.

### Behavioral analysis

The video recordings were analyzed using the Ethowatcher software (Junior et al., 2012) by a researcher blinded to the experimental manipulations: the level of spatial displacement (HD vs. LD) and the label of the objects (stationary or displaced objects). We considered as object exploration time intervals when a rat faced an object at least 2 centimeters away from its snout for more than 1 s. In order to reduce locomotion-related modulation of hippocampal oscillations, the following analyses have only included behavioral epochs of object exploration in which the animals were clearly still and not running or walking. The total exploration time was then calculated as the sum of the time spent exploring each object cumulatively minute-by-minute within each 5-min session (sample, HD and LD tests), similarly to previous studies (Dix and Aggleton, 1999; Ameen-Ali et al., 2015; Araujo et al., 2021). In order to test novel object location recognition memory, we used a discrimination index that evaluates the animal’s spontaneous preference for one of the objects. The discrimination index measure was calculated as the ratio between the time spent exploring the displaced object (tDO) minus the stationary object (tSO) and the sum of the time spent exploring both objects (tDO + tSO) in a cumulative minute-by-minute approach [(tDO − tSO)/(tDO + tSO)] (Ennaceur and Delacour, 1988; Inostroza et al., 2013; Cohen and Stackman, 2015). Positive discrimination index values indicate a preference for the displaced object, negative values indicate a preference for the stationary object and zero denotes no preference.

### Electrophysiological analysis

Signal analyses were made using custom-made and built-in routines in MATLAB (MathWorks). At first, the raw electrophysiological signals from the 16 electrodes were downsampled from 20 to 1 kHz in order to obtain the local field potentials (LFP). To do that, we used the “resample” function from the Signal Processing Toolbox, which avoids aliasing effects. We then selected one electrode from each hippocampal area per hemisphere based on differences in the phase of theta oscillations, similarly to Scheffer-Teixeira et al. (2012). Specifically, we filtered the LFP in the theta (6–12 Hz) band using the “eegfilt” function from the EEGLAB Toolbox (Delorme and Makeig, 2004). We then calculated the Hilbert transform using the “hilbert” function from the Signal Processing Toolbox to obtain the instantaneous theta phase of each LFP, from which we calculated the theta phase difference between the most superficial electrode and all other electrodes from the same hemisphere. Since theta phase reversal is known to occur between CA1/CA3 areas and the DG close to the stratum radiatum and stratum lacunosum-moleculare (Brankack et al., 1993; Buzsáki, 2002; Csicsvari et al., 2003), we selected CA1 electrodes that showed the lowest phase difference, DG electrodes that showed the highest phase difference (i.e., phase reversal), and CA3 electrodes that showed the lowest phase difference relative to the theta phases exhibited by the most superficial electrode. Electrode positioning was confirmed by the profile of power in the theta and slow gamma (25–55 Hz) bands (Supplementary Figure 3), which peak at the hippocampal fissure and at the hilus of the DG, respectively (Brankack et al., 1993; Bragin et al., 1995; Buzsáki, 2002; Csicsvari et al., 2003).

Next, LFP signals from epochs of object exploration were concatenated into a single continuous string of data for each area (Sabolek et al., 2009) and labeled according to each animal, object identity and displacement condition. Two experimental conditions were directly compared: (1) epochs of object exploration during both HD and LD test conditions, and (2) epochs of exploration of stationary and displaced objects within HD test condition since animals only discriminated between stationary and displaced objects in this test session.

**FIGURE 3:**
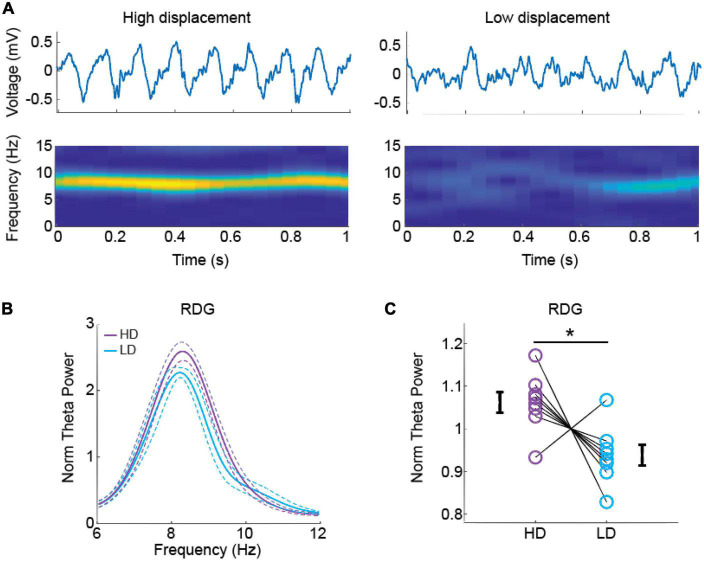
Theta oscillations in the dentate gyrus (DG) during object exploration in the high and low spatial displacement tests. **(A)** Representative raw local field potentials (LFP) (upper) and spectrograms (lower) from the right DG during 1-s of object exploration in the high and low spatial displacement tests. **(B)** Normalized average power spectra at the theta (6–12 Hz) band in the right DG during high and low spatial displacement tests (purple and cyan, respectively). Solid lines represent mean and dashed lines represent SEM. **(C)** Normalized mean theta power in right DG area at high and low spatial displacement tests. Purple and cyan circles represent normalized theta power from individual rats and error bars represent SEM. Asterisk indicates statistical significance in a paired *t* test, *n* = 8 animals.

**FIGURE 4:**
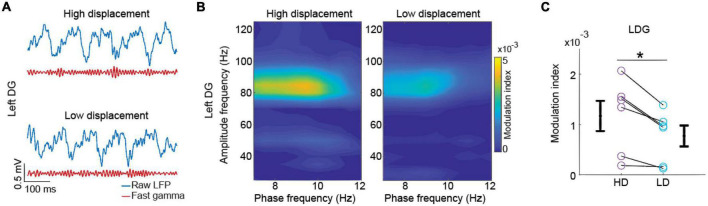
Phase-amplitude cross-frequency coupling between theta and fast gamma oscillations in the dentate gyrus during high displacement (HD) and low displacement (LD) tests. **(A)** Raw local field potentials (LFP) signals (blue) and their respective fast gamma-filtered components (red) obtained from the left DG during object exploration in the high and low spatial displacement tests (upper and lower, respectively). **(B)** Representative theta-gamma phase-amplitude modulation in the left DG during object exploration in high and low displacement tests (left and right, respectively). **(C)** Average modulation index between theta phases and fast gamma amplitude in the left DG during high and low displacement tests (purple and cyan, respectively). Circles represent modulation index for individual rats and error bars represent SEM. Asterisk indicates statistical significance in a paired *t* test, n = 6 animals.

We analyzed the power spectra at the theta (6–12 Hz), slow gamma (25–55 Hz), and fast gamma (65–110 Hz) band frequencies (Buzsáki and Draguhn, 2004; Zheng et al., 2016). We used the “spectrogram” function (0.5-s window, with 50% overlap) to obtain the time-frequency decomposition of LFP signals shown in Figures 3, 4. We used the “pwelch” function (1-s window, with no overlap) to obtain the power spectral density of LFPs. The power at a given frequency band was defined as the mean of the power spectral values within the band of interest. The power of each frequency band was then averaged across animals and test conditions to obtain the mean power of the group in HD and LD test conditions.

**FIGURE 5:**
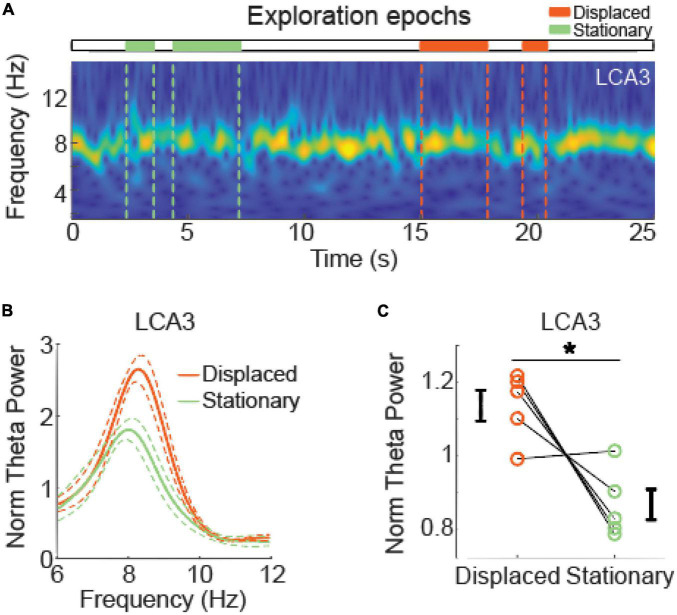
Theta oscillations in CA3 during exploration of displaced and stationary objects in the high displacements (HD) tests. **(A)** Representative spectrogram of local field potentials (LFP) signals from the left CA3 area during a high displacement test. Horizontal bars and vertical dashed lines depict time intervals of exploration of displaced and stationary objects (orange and green, respectively). **(B)** Normalized average power spectra at the theta (6–12 Hz) band in the left CA3 during exploration of displaced and stationary objects in HD tests. Solid lines represent mean and dashed lines represent SEM. **(C)** Normalized mean theta power in the left CA3 during exploration of displaced and stationary objects. Circles represent mean theta power of individual rats and error bars represent SEM. Asterisks represent statistical significance in a paired *t* test, *n* = 5 animals.

To evaluate the phase-amplitude cross-frequency coupling (CFC), the modulation index was calculated as previously described by Tort et al. (2008). Briefly, the modulation index for several frequency pairs of low-frequency “phase-modulating” and high-frequency “amplitude-modulated” components was evaluated. We first filtered spectral components of the LFPs in the theta and gamma bands. Phase bandwidth of 4 Hz at 0.5-Hz-steps were used to obtain the phases of theta oscillations between 5 and 10 Hz, and amplitude bandwidth of 10 Hz at 5-Hz-steps were used to obtain the gamma amplitude between 20 and 120 Hz. We next calculated the Hilbert transform to obtain the instantaneous phase of theta oscillations and the instantaneous amplitude of gamma oscillations. The modulation index was obtained for each electrode and experimental condition individually. To obtain the CFC between theta phases and a given subcomponent of the gamma frequency band, we averaged the modulation index within the slow and fast gamma band frequencies previously defined. We thus compared modulation index values between HD and LD conditions. No CFC analysis was performed to compare phase-amplitude modulation during the exploration of stationary and displaced objects due to short epochs of contact with objects for two animals, i.e., the total time of contact with one of the objects was lower than 1.5 s. Modulation index values were graphically expressed as color-coded plots (Figure 5), in which hot colors in the c-axis indicate that the phase-frequency in the x-axis modulates the amplitude-frequency in the y-axis.

In order to reduce variability among animals due to differences in electrode impedance, electrode position, and other factors, statistical comparisons of band power were performed after the normalization of individual data points (animal and condition) by the mean across conditions (Tort et al., 2009; Belchior et al., 2014; Furtunato et al., 2020). For instance, in the power spectral analysis (Figures 3B, 4B), the theta band power in HD condition was divided by the mean power across HD and LD conditions Normalized HD = HD/[(HD + LD)/2]. The same normalization was applied to LD conditions before statistical comparison. Thus, the sum of power in HD and LD conditions after normalization must be equal to one.

### Statistical analysis

MATLAB (MathWorks) and SPSS (v.26, IBM) were used for statistical analyses, and results were considered significant at an α level lower than 0.05. SPSS was used for behavioral analyses, while MATLAB was used for electrophysiological analyses and their correlations with behaviors. The Shapiro-Wilk test was used to analyze data normality in both behavioral and electrophysiological datasets. For behavioral analyses, the two-way ANOVA followed by the Sidak-Bonferroni *post-hoc* test was used to compare the total exploration time across time and conditions (minute-by-minute and high and low spatial displacement tests, respectively). The Wilcoxon signed-rank test was used to compare the discrimination indexes against chance levels (i.e., no preference: 0), and also to directly compare the discrimination indexes between conditions of HD and LD. The paired *t* test was used to compare band power values between exploration time in HD and LD test conditions and between exploration of objects in displaced and stationary conditions. The “corr” function was used to evaluate the Spearman’s rank order correlation (rho) between the discrimination index and the power of theta, slow gamma and fast gamma bands. We used the GPower software to calculate Cohen’s (d’) effect size (Faul et al., 2009), in which we considered d’ values > 0.8 as a large effect size.

## Results

### Rats discriminate between stationary and displaced objects in the high displacement test

Rats executed an object recognition task with high and low spatial displacement test (HD: 108 and LD: 54 cm; see Figure 2A). During the sample session, no significant difference was found neither in the total time exploring the two objects [Figure 2B, t(7) = 0.154, *p* = 0.882, *d*’ = 0.055, paired *t* test] nor in the preference for specific objects [i.e., discrimination index against 0; Figure 2C, t(7) = 0.077, *p* = 0.941, *d*’ = 0.696, Wilcoxon signed-rank test]. During the tests, the total exploration times in HD and LD conditions were not statistically different (Figure 2D). Two-way ANOVA, in a minute-by-minute cumulative analysis, detected no differences between the task conditions [HD x LD, F(1,27) = 2.836, *p* = 0.104, *d*’ = 0.323] nor detected differences for factor interaction [F(4,108) = 2.348, *p* = 0.120, *d*’ = 0.294], which suggests equivalent motivational drive to explore the objects in both HD and LD tests. Also, a direct comparison of the discrimination index in HD and LD conditions revealed no significant difference (*p* = 0.089, *d*’ = 0.287, Wilcoxon signed-rank test). In spite of that, rats exhibited an exploration preference for the displaced object in opposition to the stationary object in the HD test, as shown by the discrimination index statistically higher than chance in minutes 2 (Figure 2E, *p* = 0.048; *d*’ = 1.005) and 3 (*p* = 0.048; *d*’ = 0.971); while minutes 1 (*p* = 0.105; *d*’ = 1.798), 4 (*p* = 0.061; *d*’ = 0.825), and 5 (*p* = 0.061; *d*’ = 0.825) were not significantly different from chance (Wilcoxon signed-rank test against 0). In contrast, the discrimination index in LD tests was not statistically different from zero (see Supplementary Table 1). These behavioral results suggest that rats do discriminate between stationary and displaced objects in conditions of pronounced spatial changes, but not in smaller spatial change conditions.

In order to investigate whether oscillatory activity in the hippocampus is associated with the discrimination of stationary and displaced objects–either in small or pronounced spatial changes - we bilaterally recorded local field potentials from CA1, CA3, and DG areas using multielectrode arrays (Figure 1A, left and middle). Histological analysis confirmed electrode tip positions at the dorsal hippocampus (Figure 1A, right). We then used the theta phase reversion between LFP signals from DG and CA1/CA3 to select one electrode from each subfield in both hemispheres (Supplementary Figure 3). Figure 1B (and Supplementary Figure 3) shows representative raw local field potentials during 1-s of rhythmic activity in the theta (6–12 Hz) band obtained from the left hemisphere of the hippocampus. Panels C and D of the Supplementary Figure 3 show the laminar profile of theta power and slow gamma power across the dorsal hippocampus.

### Recognition memory was associated with higher theta power in the dentate gyrus

Subsequently, we next investigated whether there would be any differences in oscillatory LFP activity during the retrieval/test phase between the condition animals detected the displaced object (HD tests) and the condition animals did not discriminate between displaced and stationary objects (LD tests). Raw LFPs and spectral decompositions during object exploration epochs exhibited stronger theta rhythm in the HD test in comparison to the LD test (Figure 3A, left and right panels respectively). The group result shows that the normalized theta power in the right DG was also statistically higher in the HD test [Figures 3B, C, RDG, t(7) = 2.576, *p* = 0.036, *d*’ = 0.911]; no significant difference was observed in the left DG. We found no statistical difference in theta power between HD and LD conditions neither in CA3 nor CA1 areas. We found no significant difference between HD and LD conditions neither for the slow gamma (25–55 Hz) nor fast gamma (65–110 Hz) bands. Supplementary Table 2 shows statistical results for power spectra comparisons between HD and LD conditions in the theta, slow and fast gamma bands.

### Theta-fast gamma phase-amplitude coupling in the dentate gyrus was higher in HD than LD tests

We also evaluated whether the theta phase modulates the amplitude of gamma oscillations during object contacts and whether it changes between different memory conditions in HD and LD tests. A representative example of theta-phase-associated gamma burst in the left DG is shown in Figure 5A. Comodulograms from the left DG show that in both HD and LD conditions the modulation index peaked at ∼80 Hz, within the fast gamma (65–110 Hz) band (Figure 5B). We found that the theta-fast gamma phase-amplitude coupling was significantly higher during the HD than LD tests in the left DG [Figure 5C, LDG t(5) = 3.856, *p* = 0.012, *d*’ = 1.074; paired *t* test]. We found no significant difference in the theta-gamma modulation between HD and LD in other brain areas. Supplementary Figure 4 shows comodulograms from individual rats and Supplementary Table 3 shows statistical results according to brain areas and slow and fast gamma frequency bands.

### Exploration of displaced objects was associated with higher theta power in CA3

To further investigate whether hippocampal rhythms are associated with the discrimination of objects, we compared LFPs during the exploration of displaced and stationary objects in the HD test. Spectral analysis revealed the presence of theta oscillations during exploration of both stationary and displaced objects (Figure 4A). Normalized theta band power in the left CA3 was higher during the exploration of displaced objects than stationary ones [Figures 4B, C, LCA3 t(4) = 3.250, *p* = 0.031, *d*’ = 1.181, paired *t* test]. We found no significant differences between stationary and displaced objects in theta band power in the right CA3, nor in DG and CA1 of both hemispheres; we found no significant differences in the slow and fast gamma band power in none of the areas. Supplementary Table 4 shows statistical results of spectral power according to brain areas and frequency bands.

### The discrimination index was correlated with gamma band power in CA1

**FIGURE 6:**
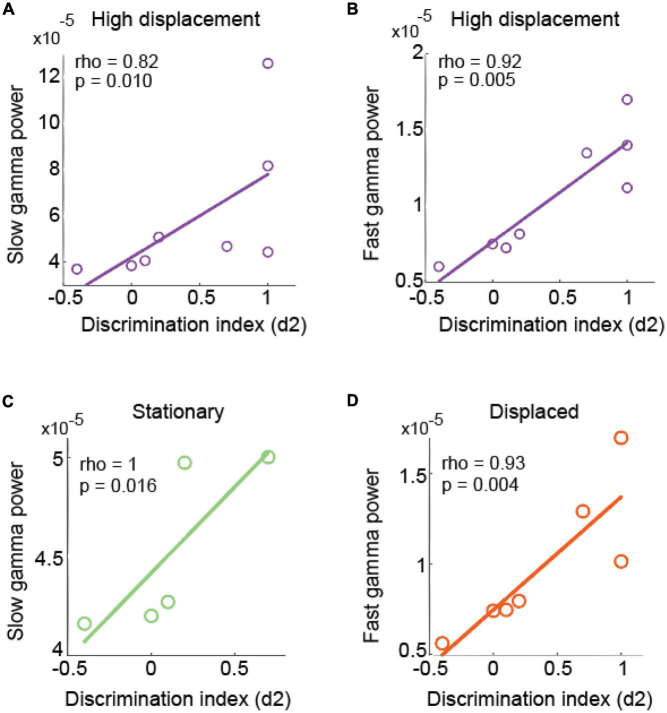
Relationship between the discrimination index and the power of slow and fast gamma bands in CA1 during exploration of displaced and stationary objects in high displacements (HD) tests. Relationship between the discrimination index and the power of slow gamma **(A)** and fast gamma **(B)** bands exhibited in the right CA1 during object exploration in HD tests. Circles represent individual animals, and the straight line depicts the linear relationship between variables. **(C)** Relationship between the power at slow gamma band exhibited during exploration of stationary (green) objects and the discrimination index in HD tests. **(D)** Relationship between the power at fast gamma band exhibited during exploration of displaced (orange) objects and the discrimination index in HD tests. Panels **(A–D)** represent 8, 5 and 7 animals, respectively.

We next analyzed the relationship between the discrimination index and the power of hippocampal theta, slow and fast gamma oscillations during object exploration epochs. We found no significant relationship between the discrimination index and theta, slow or fast gamma band power in the LD tests. Nonetheless, the discrimination index positively correlated with the gamma band power in the right CA1 area. Both slow and fast gamma band power exhibited during object exploration were significantly correlated with the discrimination index (Figures 6A, B, rho = 0.829, *p* = 0.016; rho = 0.927, *p* = 0.005, respectively). No significant correlation was observed between the discrimination index and theta, slow or fast gamma in other brain areas (see Supplementary Table 5).

We also evaluated the correlation between the discrimination index and theta, slow and fast gamma band power specifically obtained during exploration of displaced and stationary objects within the HD tests. The discrimination index positively correlated with slow gamma power in the right CA1 exhibited during exploration of stationary objects (Figure 6C, rho = 1, *p* = 0.016). Moreover, the discrimination index was positively correlated with fast gamma band power in the right CA1 during exploration of the displaced object (Figure 6D, rho = 0.936, *p* = 0.004). Of note, the discrimination index was also inversely correlated with the theta band power in the right DG during the exploration of the stationary object (rho = −1, *p* = 0.016; Supplementary Tables 6, 7).

## Discussion

We employed an object recognition task and multielectrode recordings from the rat hippocampus to investigate the electrophysiological correlates of the recognition memory for spatial displacements of objects by large and small distances. Our results show that rats do discriminate between stationary and displaced objects in conditions of pronounced displacement (HD, 108 cm) but not low displacement (LD, 56 cm), which allowed us to directly compare between different behavioral outcomes in the retrieval phase of the test. Spectral analysis of the LFP activity revealed (1) prominent theta oscillations during epochs of contact with the objects, (2) higher theta power in the right DG during HD than LD tests, (3) higher theta-gamma phase-amplitude coupling in the left DG during HD than LD tests. In addition, (4) contacts with displaced objects exhibited higher theta power in the left CA3 than stationary objects in the HD tests. Finally, (5) the discrimination index directly correlated with gamma band power in the right CA1 during object contacts, in which slow gamma oscillations related to exploration of stationary objects (i.e., memory retrieval) and fast gamma oscillations related to displaced objects (encoding). In all, these findings suggest that the theta and gamma oscillatory activity in the dorsal hippocampus is positively related to object discrimination in a recognition memory task.

Recent studies have used recognition memory tasks in rodents to investigate the discrimination of spatially displaced and stationary objects, as well as the underlying processing of memory encoding and retrieval in hippocampal circuits. Hunsaker and Kesner (2008) found that chemical lesions in the rat DG impairs the discrimination of previously encountered objects in conditions of low but not high spatial displacements, suggesting that the DG processing is critical to detect fine spatial displacements. In their experiment, sham-lesioned rats (control group) exhibited significant discrimination index scores in both high (108 cm) and low (56 cm) spatial displacement tests (Hunsaker and Kesner, 2008). Contrasting to that, we have found that rats were only capable of discriminating between stationary and displaced objects–i.e., discrimination indexes higher than chance - in large displacement conditions (108 cm). Such contrast allowed for the comparison between different memory outcomes and their underlying mechanisms.

Since the task protocols in both studies followed similar displacement conditions, we attribute this behavioral difference to divergences in the amount of sampling/test phases: while Hunsaker and Kesner (2008) used three sampling phases followed by one test, here we used only one sampling phase followed by two tests. It might be the case that task designs with multiple sample trials–as used by Hunsaker and Kesner (2008)–may facilitate the acquisition of memory for the spatial location of objects in fine displacement conditions. In addition, the two studies also differed in the strain of rats used: Hunsaker and Kesner (2008) used Long Evans rats and we used Wistar strain, which could also contribute to the observed variability (Andrews et al., 1995). Nevertheless, to the best of our knowledge, this is the first time in which the successful discrimination of displaced objects was associated with high but not low spatial displacements in rats (but see also Reichelt et al., 2021).

We then evaluated LFP activity in the dorsal hippocampus by comparing among behavioral conditions of successfull discrimination and no explicit behavioral expression of object discrimination - observed in the HD and LD test conditions, respectively. Our electrophysiological results associated increases in theta band power in the right DG to the effective discrimination of objects in conditions of pronounced spatial changes. In contrast, no similar changes in theta band power were observed contralaterally in the left DG nor in CA3 or CA1 areas of both hemispheres. These results are in line with previous studies on hippocampal lesions, which suggest a pivotal role of the DG in the detection of spatial displacements of objects (Gilbert et al., 2001; Hunsaker and Kesner, 2008). Furthermore, other studies reported that the optogenetic silencing and pharmacological inactivation of the DG also impairs the discrimination of displaced objects in recognition memory tasks (Barbosa et al., 2012; Fernández-Ruiz et al., 2021) and during the discrimination of aversive stimuli in a spatial memory task (van Dijk and Fenton, 2018).

Our results of cross-frequency coupling also highlighted the role of the DG in the processing of recognition memory, which revealed a stronger phase-amplitude modulation between theta and fast gamma oscillations in the left DG during object exploration in HD than LD test conditions. These results paralel those of Tort et al. (2009) that found theta-slow gamma phase-amplitude modulation in CA3 during an odor-place discrimination task. The authors also found that the levels of theta-slow gamma modulation were positively correlated with memory performance. Others have associated theta-slow gamma modulation in CA1 with the successful encoding of object identity (Trimper et al., 2014). On the other hand, Fernández-Ruiz et al. (2021) reported that MEC-DG projections sustain theta-fast gamma coupling during an object-place recognition memory task, which was affected by the optogenetic perturbation of MEC. Taken together, these findings suggest that the dynamic modulation of gamma amplitude by the phases of theta oscillations throughout the hippocampus-entorhinal axis may support recognition memory. Future studies could test whether the optogenetic or chemogenetic disruption of theta oscillations or theta-gamma phase-amplitude coupling specifically during object exploration impairs performance in recognition memory tasks.

Since recognition memory tasks allow the analysis of object-associated brain activity under very similar behavior conditions, we compared theta oscillations during the exploration of stationary and displaced objects in conditions of explicit discrimination. We observed that the left CA3 expressed stronger theta power during the exploration of displaced objects at HD test condition, suggesting an involvement of CA3 theta oscillations in the detection of a new position of the familiar object. Using a NOL recognition memory task, Zheng et al. (2016) found no changes in CA3 theta power when directly comparing displaced and stationary objects. However, it may be due to the fact that in their task animals did not explicitly discriminate between object conditions in the probe session. As far as we know, no other study reported changes in CA3 theta power due to exploration of displaced and stationary objects.

In parallel to analyzing theta oscillations, Zheng et al. (2016) reported that CA1 expressed increased fast gamma band power when rats explored a new object in a new place, and suggested that fast gamma oscillations may encode new associations between place and object identity. Trimper et al. (2017) also found that slow gamma band power and coherence among DG and CA3 were associated with performance in a novel object and object-location memory task. In opposition to that, here we found no significant changes in gamma band power between high and low displacement test conditions, nor statistical changes between stationary and displaced objects. Instead, we have found a positive relationship between gamma oscillations and the discrimination index when explicit recognition memory was detected (HD test condition). In addition, the discrimination index was positively correlated with the power of both CA1 slow and fast gamma bands during the exploration of objects. Moreover, slow gamma band power was particularly associated with the exploration of stationary objects (memory retrieval). On the other hand, fast gamma band power was associated with the exploration of displaced objects (memory encoding). These results corroborate previous findings showing that slow gamma oscillations may route information from CA3 to CA1 supporting memory retrieval, while fast gamma allows direct communication between the medial entorhinal cortex and CA1, supporting memory encoding (Colgin et al., 2009; Colgin, 2016).

Our results revealed an apparent asymmetry between hippocampal hemispheres, since we found significant differences in theta band power between HD and LD conditions only in the right hemisphere and significant differences in theta-fast gamma phase-amplitude coupling only in the left hemisphere. Although some studies investigated hippocampus asymmetry (Shipton et al., 2014; Song et al., 2020; Guan et al., 2021), it is still unclear how lateralized functions could affect memory processes. It has been suggested that both left and right CA3 are involved in short-term memory, while left CA3 is essential on a long-term spatial memory task (Shipton et al., 2014). However, Song et al. (2020) found an involvement of left CA3 in a spatial working memory task. To the best of our knowledge, no studies have addressed the role of hippocampal lateralization in object recognition tasks, so at this point it is precocious to conclude whether interhemispheric asymmetry has functional importance. Future studies are needed to answer this issue.

Overall, we believe that our results are consistent with the notion that the processing of mnemonic information is supported by theta and gamma oscillatory activity in the rat hippocampus (Tort et al., 2009; Belchior et al., 2014; Colgin, 2016; Fernández-Ruiz et al., 2021). Theta and gamma oscillations are thought to foster memory encoding and retrieval providing temporal windows for effective neuronal communication and spike-timing neuronal plasticity in hippocampal circuits and associated areas (Markram et al., 1997; Buzsáki, 2002; Fries, 2005). Therefore, our results highlight the function of different hippocampal areas on the discrimination of displaced and stationary objects, in which theta and gamma rhythms may play a critical role in the detection of spatial changes in recognition memory tasks.

## Data availability statement

The raw data supporting the conclusions of this article will be made available by the authors, without undue reservation.

## Ethics statement

This animal study was reviewed and approved by Ethics Committee on the Use of Animals (CEUA/UFRN, permit n° 52/2016).

## Author contributions

FB, BL-S, and HB designed the study. LN, AF, IP, NS, AM, GN, and EG collected the data. LN, AA, and HB analyzed the data. FB, BL-S, AT, and HB wrote the manuscript. All authors contributed to the article and approved the submitted version.
