# Supplementary material for: Theta and gamma oscillations in the rat hippocampus support the discrimination of object displacement in a recognition memory task
Source: Front Behav Neurosci. 2022 Dec 21;16:970083. doi: 10.3389/fnbeh.2022.970083 (PMC9811406; doi:10.3389/fnbeh.2022.970083)
Supplement: Supplementary file 1 [file Data_Sheet_1.docx]

**SUPPLEMENTARY MATERIALS**

**
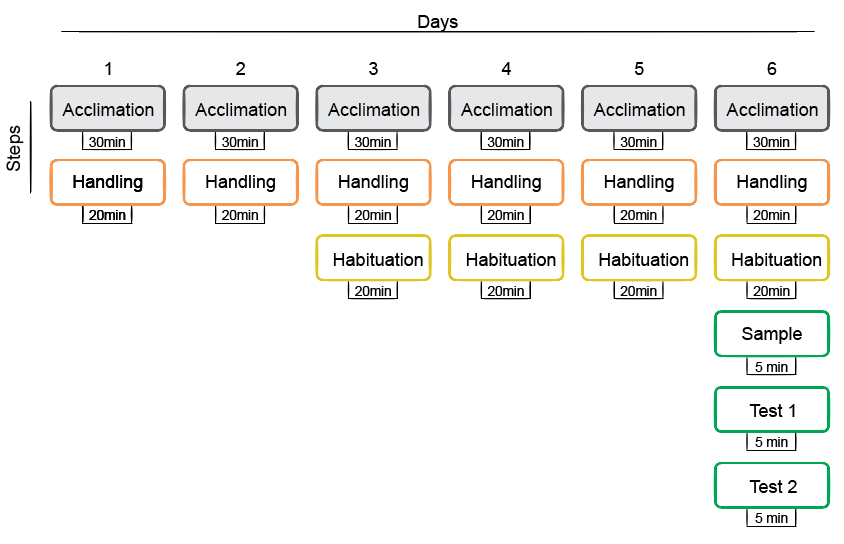
**

**Supplementary Figure 1 - Protocol design showing periods of *Acclimation*, *Handling*, *Habituation*, and the *Object recognition task***. The object recognition task consisted of a 5-minute sample trial, followed by two 5-minute test trials. The inter-trial interval was 10 minutes. The order of the two test trials (Test 1 and 2) was balanced among animals to start with either high or low spatial displacement manipulations.


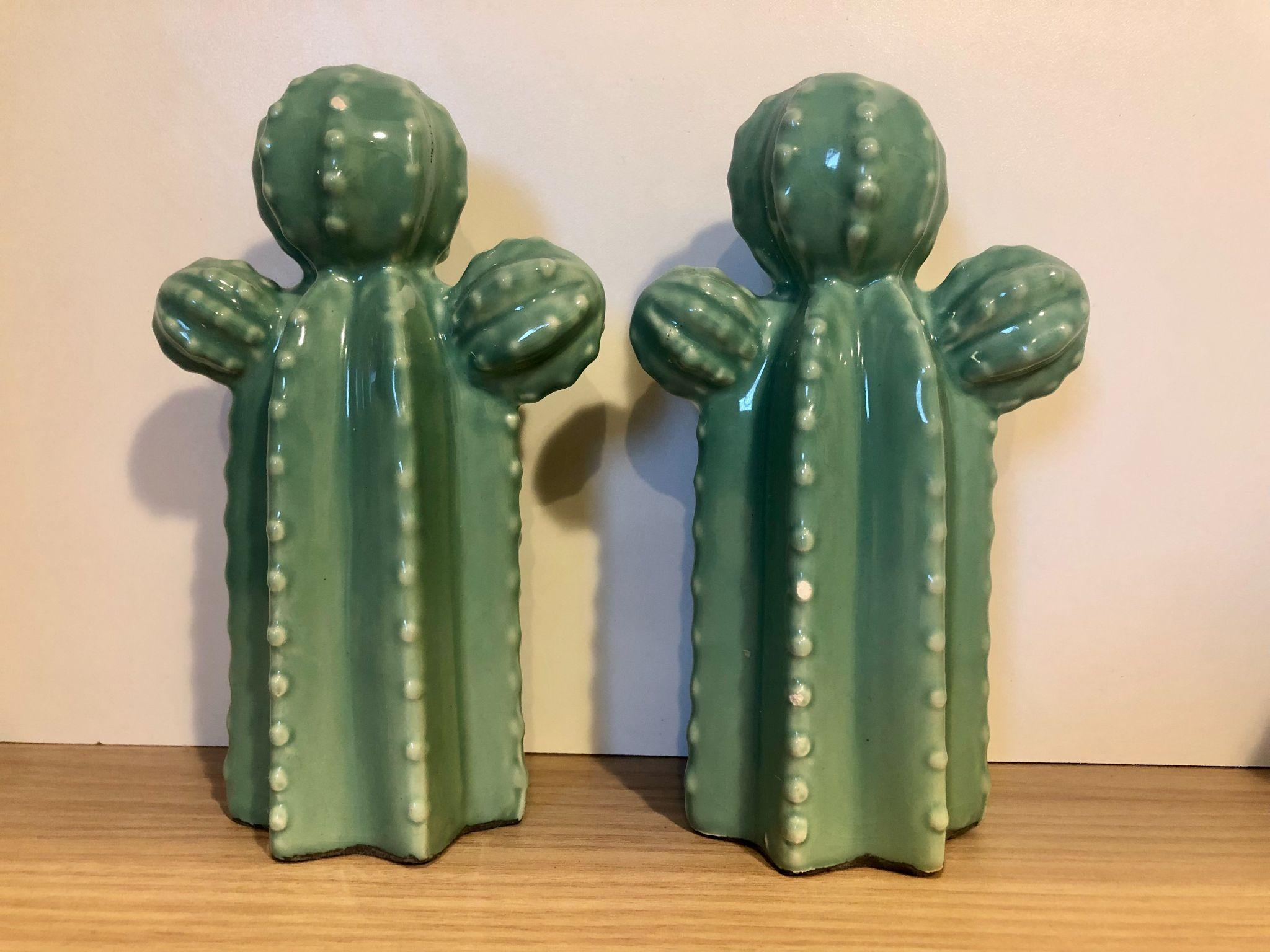


**Supplementary Figure 2** - **Illustrative picture of the two identical porcelain objects used in the object recognition task.** Object dimensions were: 16.5 cm, 9.5 cm, 6 cm (H. x L. x W., respectively).


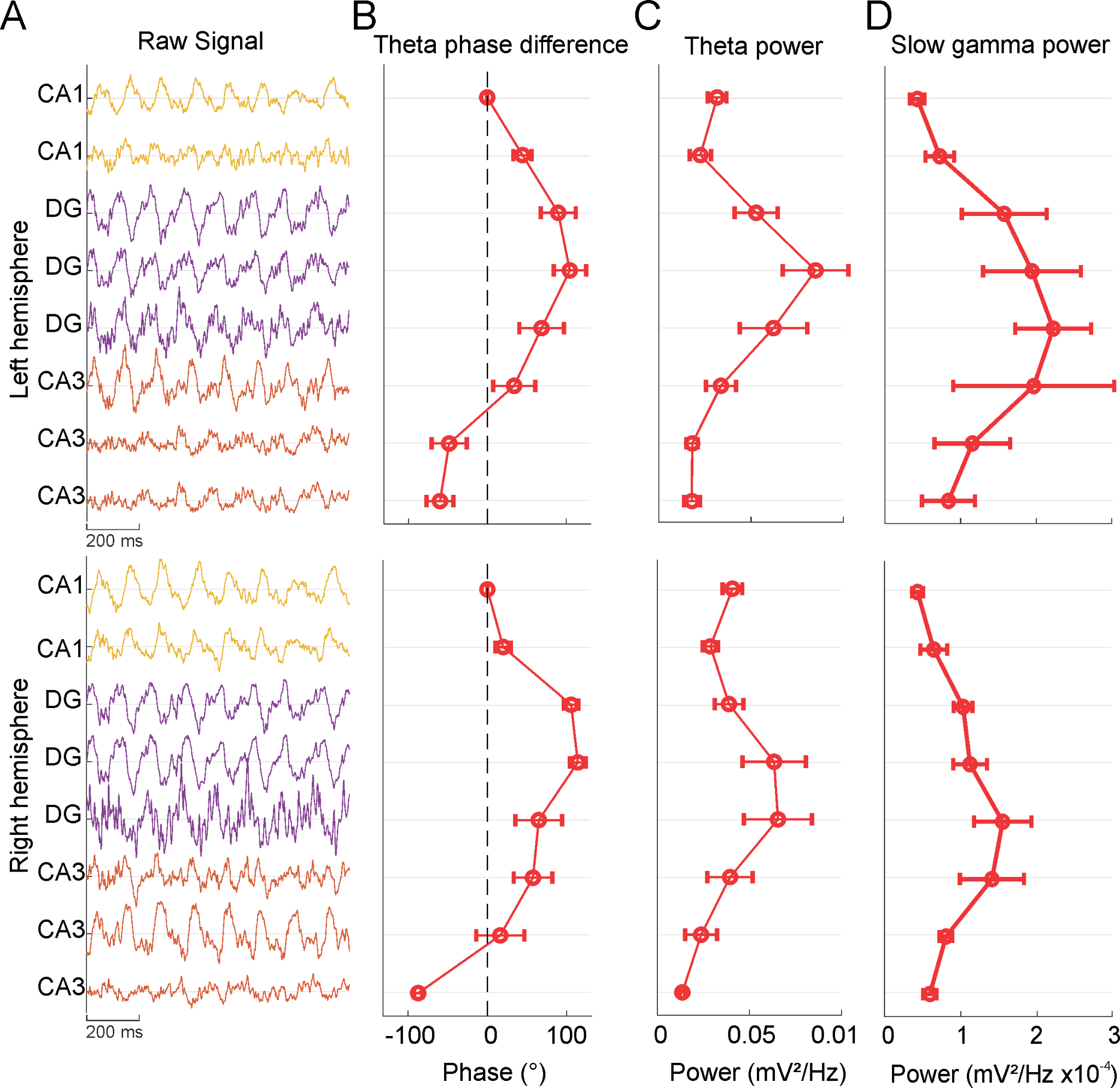


**Supplementary Figure 3 - Laminar profile of the theta phase difference, theta band power, and slow gamma band power across electrodes at the left and right hemispheres.** (A) Raw LFP signals from two electrodes at CA1, three electrodes at DG and three electrodes at CA3 from the left (upper) and right (lower) hemispheres. (B) Theta phase differences between electrodes pairs at CA1, DG and CA3 from the left (upper) and right (lower) hemispheres. Circles denote mean band power across animals, error bars denote SEM. (C) Theta band power across electrodes at CA1, DG and CA3 from the left (upper) and right (lower) hemispheres. (D) Slow gamma power from electrodes at CA1, DG and CA3 from the left (upper) and right (lower) hemispheres.


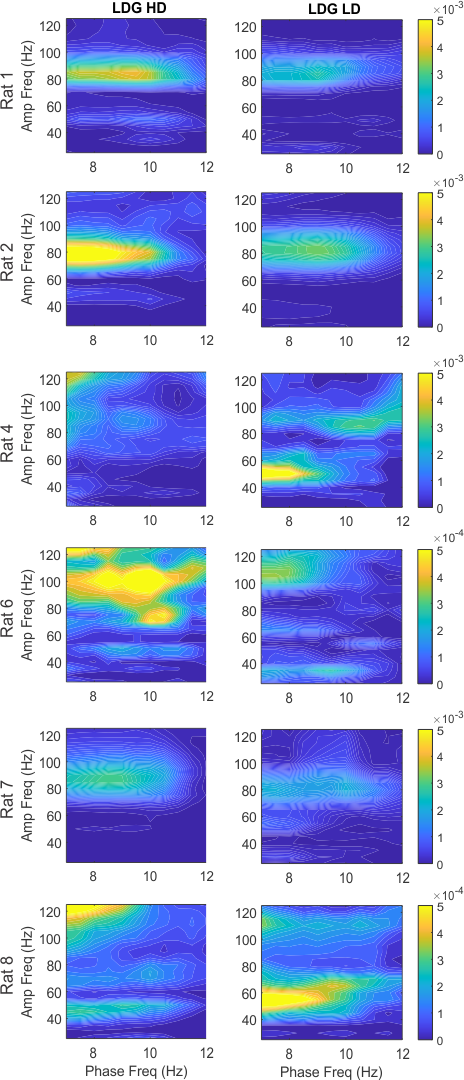


**Supplementary Figure 4** - **Theta-gamma phase-amplitude modulation in the left DG in the HD and LD conditions across all animals.** The first column shows modulation during object exploration in HD condition, and the second column in LD condition. The modulation index is color coded, as shown in the legend bar. Lines represent individual animals (n = 6 animals).

|  | **Test conditions** | | | | | | |
| --- | --- | --- | --- | --- | --- | --- | --- |
| **Cumulative time** | **HD** | | |  | **LD** | | |
|  | **p-value** |  | **U-value** |  | **p-value** |  | **U-value** |
| Minute 1 | 0.105 |  | 1.620 |  | 0.523 |  | 0.639 |
| Minute 2 | **0.048*** |  | 1.980 |  | 0.526 |  | 0.634 |
| Minute 3 | **0.048*** |  | 1.980 |  | 0.291 |  | 1.057 |
| Minute 4 | 0.061 |  | 1.873 |  | 0.325 |  | 0.985 |
| Minute 5 | 0.061 |  | 1.873 |  | 0.360 |  | 0.916 |

**Supplementary Table 1 - The discrimination index against chance levels in a minute-by-minute cumulative way during HD and LD test conditions.** The first column shows the cumulative time across five minutes. Subsequent columns show statistical results using the Wilcoxon signed-rank test for the discrimination index against zero in HD and LD test conditions. The statistical U-values and p-values are shown for each condition, significant p-values are shown in bold with an asterisk.

|  |  |  |  |  |  | **Means** | |  |  |
| --- | --- | --- | --- | --- | --- | --- | --- | --- | --- |
| **Frequency band** | **Area** | **t-value** |  | **p-value** |  | **HD** | **LD** |  | **STD** |
| **Theta** | **LCA1** | 0.419 |  | 0.687 |  | 1.017 | 0.983 |  | 0.106 |
|  | **RCA1** | 1.437 |  | 0.194 |  | 1.030 | 0.970 |  | 0.054 |
|  | **LCA3** | 0.707 |  | 0.502 |  | 1.038 | 0.962 |  | 0.140 |
|  | **RCA3** | 1.045 |  | 0.331 |  | 1.040 | 0.960 |  | 0.101 |
|  | **LDG** | 2.301 |  | 0.055 |  | 1.056 | 0.944 |  | 0.063 |
|  | **RDG** | 2.577 |  | **0.037*** |  | 1.061 | 0.938 |  | 0.063 |
| **Slow Gamma** | **LCA1** | 0.301 |  | 0.772 |  | 1.011 | 0.989 |  | 0.095 |
|  | **RCA1** | 1.437 |  | 0.506 |  | 0.978 | 1.022 |  | 0.082 |
|  | **LCA3** | 0.851 |  | 0.423 |  | 0.967 | 1.032 |  | 0.100 |
|  | **RCA3** | 0.239 |  | 0.818 |  | 1.007 | 0.992 |  | 0.078 |
|  | **LDG** | 0.657 |  | 0.532 |  | 0.977 | 1.022 |  | 0.090 |
|  | **RDG** | 1.011 |  | 0.346 |  | 0.971 | 1.029 |  | 0.076 |
| **Fast Gamma** | **LCA1** | 0.506 |  | 0.628 |  | 0.986 | 1.013 |  | 0.070 |
|  | **RCA1** | 0.420 |  | 0.687 |  | 1.01 | 0.990 |  | 0.062 |
|  | **LCA3** | 0.984 |  | 0.358 |  | 0.958 | 1.042 |  | 0.113 |
|  | **RCA3** | 1.103 |  | 0.306 |  | 1.032 | 0.968 |  | 0.076 |
|  | **LDG** | 0.262 |  | 0.801 |  | 0.991 | 1.009 |  | 0.094 |
|  | **RDG** | 1.243 |  | 0.254 |  | 1.028 | 0.972 |  | 0.058 |

**Supplementary Table 2 - Paired t tests of power spectra between high and low displacement tests.** The first and second column show frequency bands from the hippocampal areas (L: Left and R: Right) respectively. Subsequent columns show statistical results using paired t tests for comparison between two HD and LD test conditions: the t-value, p-value, and the means for high displacement (HD) and low displacement (LD) tests, followed by the correspondent standard deviation (STD). The statistical t-values and p-values are shown for each area, significant p-values are shown in bold with an asterisk.

| **Frequency band** |  |  |  |  | | |  | **Means (STD)** | | |
| --- | --- | --- | --- | --- | --- | --- | --- | --- | --- | --- |
|  |  | **Area** |  | **t-value** |  | **p-value** |  | **HD** |  | **LD** |
| **Theta-Slow Gamma** |  | **L CA1** |  | 1.007 |  | 0.360 |  | 0.0003  (0.00042) |  | 0.0007 (0.00094) |
|  |  | **R CA1** |  | 0.688 |  | 0.522 |  | 0.0002  (0.000125) |  | 0.0006  (0.000648) |
|  |  | **L CA3** |  | 0.046 |  | 0.965 |  | 0.0003  (0.00035) |  | 0.0003  (0.00031) |
|  |  | **R CA3** |  | 0.993 |  | 0.366 |  | 0.0002  (0.00020) |  | 0.0002  (0.00025) |
|  |  | **L DG** |  | 0.809 |  | 0.455 |  | 0.0002  (0.00013) |  | 0.0005  (0.00073) |
|  |  | **R DG** |  | 1.071 |  | 0.333 |  | 0.0003  (0.00019) |  | 0.0003 (0.00036) |
| **Theta-Fast Gamma** |  | **L CA1** |  | 0.709 |  | 0.510 |  | 0.0005 (0.00025) |  | 0.0008  (0.00092) |
|  |  | **R CA1** |  | 0.741 |  | 0.492 |  | 0.0006  (0.00043) |  | 0.0009  (0.00105) |
|  |  | **L CA3** |  | 0.768 |  | 0.477 |  | 0.0003 (0.0001) |  | 0.0006  (0.00090) |
|  |  | **R CA3** |  | 0.388 |  | 0.714 |  | 0.0003 (0.00026) |  | 0.0006  (0.00083) |
|  |  | **L DG** |  | 3.856 |  | **0.012*** |  | 0.0012 (0.00067) |  | 0.0009  (0.00060) |
|  |  | **R DG** |  | 1.450 |  | 0.333 |  | 0.0010 (0.00085) |  | 0.0006  (0.00040) |

**Supplementary Table 3 - Paired t tests considering phase-amplitude cross-frequency coupling modulation indexes between high and low displacement tests.** The first and second column show frequency bands from the hippocampal areas (L: Left and R: Right) respectively. Subsequent columns show statistical results using paired t test for comparison between two HD and LD test conditions: the t-value and p-value, followed by the correspondent the means (first line) and standard deviation (STD, second line) for high displacement (HD) and low displacement (LD) tests. The statistical t-values and p-values are shown for each area, significant p-values are shown in bold with an asterisk.

|  |  |  |  |  |  | **Means (STD)** | |
| --- | --- | --- | --- | --- | --- | --- | --- |
| **Frequency bands** | **Area** | **t-value** |  | **p-value** |  | **Displaced** | **Stationary** |
| **Theta** | **L CA1** | 1.293 |  | 0.266 |  | 1.034(0.077) | 0.945(0.0953) |
|  | **R CA1** | 1.278 |  | 0.270 |  | 1.020(0.046) | 0.968(0.057) |
|  | **L CA3** | 3.251 |  | **0.031*** |  | 1.085 (0.100) | 0.864 (0.093) |
|  | **R CA3** | 1.978 |  | 0.119 |  | 1.081(0.130) | 0.870(0.147) |
|  | **L DG** | 1.125 |  | 0.323 |  | 1.034 (0.086) | 0.946(0.108) |
|  | **R DG** | 1.479 |  | 0.213 |  | 1.059(0.118) | 1.059(0.143) |
| **Slow Gamma** | **L CA1** | 0.769 |  | 0.485 |  | 0.985(0.05) | 1.023(0.067) |
|  | **R CA1** | 2.615 |  | 0.059 |  | 0.967(0.043) | 1.052(0.044) |
|  | **L CA3** | 1.374 |  | 0.241 |  | 0.971(0.06) | 1.045 (0.074) |
|  | **R CA3** | 1.578 |  | 0.190 |  | 0.972(0.053) | 1.045(0.064) |
|  | **L DG** | 0.339 |  | 0.751 |  | 0.992(0.058) | 1.012(0.076) |
|  | **R DG** | 0.840 |  | 0.448 |  | 0.985(0.050) | 1.024(0.064) |
| **Fast Gamma** | **L CA1** | 2.128 |  | 0.820 |  | 0.983(0.023) | 1.023(0.025) |
|  | **R CA1** | 1.787 |  | 0.148 |  | 0.960(0.063) | 1.056(0.070) |
|  | **L CA3** | 1.399 |  | 0.234 |  | 0.978(0.043) | 1.031(0.043) |
|  | **R CA3** | 0.498 |  | 0.644 |  | 1.056(0.082) | 1.056(0.100) |
|  | **L DG** | 1.141 |  | 0.318 |  | 0.979(0.048) | 0.979(0.057) |
|  | **R DG** | 0.243 |  | 0.820 |  | 0.995(0.055) | 1.007(0.068) |

**Supplementary Table 4 - Paired t test of power spectra in different frequency bands during exploration of displaced and stationary objects.** The first and second columns show frequency bands from the hippocampal areas (L: Left and R: Right), respectively. Subsequent columns show statistical results using paired t tests for comparison between HD and LD test conditions: the t-values and p-values, followed by the correspondent means and standard deviation (STD, in parenthesis) for high displacement (HD) and low displacement (LD) tests. The statistical t-values and p-values are shown for each area, significant p-values are shown in bold with an asterisk.

|  |  | **Frequency band** | | | | | | | | | | |
| --- | --- | --- | --- | --- | --- | --- | --- | --- | --- | --- | --- | --- |
|  |  | **Theta** | | |  | **Slow Gamma** | | |  | **Fast Gamma** | | |
| **Area** |  | **rho-value** |  | **p-value** |  | **rho-value** |  | **p-value** |  | **rho-value** |  | **p-value** |
| **L CA1** |  | 0.244 |  | 0.564 |  | 0.488 |  | 0.2270 |  | 0.707 |  | 0.064 |
| **R CA1** |  | -0.048 |  | 0.927 |  | 0.829 |  | **0.016*** |  | 0.902 |  | **0.005*** |
| **L CA3** |  | 0.561 |  | 0.161 |  | 0.219 |  | 0.616 |  | 0.292 |  | 0.482 |
| **R CA3** |  | -0.439 |  | 0.279 |  | -0.073 |  | 0.887 |  | 0.390 |  | 0.336 |
| **L DG** |  | 0.244 |  | 0.563 |  | 0.390 |  | 0.336 |  | 0.585 |  | 0.138 |
| **R DG** |  | -0.536 |  | 0.178 |  | 0.341 |  | 0.410 |  | 0.146 |  | 0.742 |

**Supplementary Table 5 - Spearman’s correlations between discrimination index and band power at different frequency bands during all contacts in HD condition.** The second line shows each frequency band, the first column shows hippocampal areas (L: Left and R: Right). Subsequent columns show statistical results using Spearman’s correlations between discrimination index and band power at different frequency bands: the rho-value, p-value, for for each frequency band. The statistical spearman rho, and the p-values are shown for each area and frequency band, significant p-values are in bold with an asterisk.

|  |  | **Frequency band** | | | | | | | | | | |
| --- | --- | --- | --- | --- | --- | --- | --- | --- | --- | --- | --- | --- |
|  |  | **Theta** | | |  | **Slow Gamma** | | |  | **Fast Gamma** | | |
| **Area** |  | **rho-value** |  | **p-value** |  | **rho-value** |  | **p-value** |  | **rho-value** |  | **p-value** |
| **L CA1** |  | 0.195 |  | 0.652 |  | 0.487 |  | 0.227 |  | 0.432 |  | 0.326 |
| **R CA1** |  | -0.365 |  | 0.731 |  | 0.731 |  | 0.051 |  | 0.936 |  | **0.016*** |
| **L CA3** |  | 0.512 |  | 0.205 |  | 0.219 |  | 0.616 |  | 0.108 |  | 0.822 |
| **R CA3** |  | -0.512 |  | 0.205 |  | -0.073 |  | 0.887 |  | 0.288 |  | 0.524 |
| **L DG** |  | 0.268 |  | 0.527 |  | 0.414 |  | 0.312 |  | 0.57 |  | 0.186 |
| **R DG** |  | -0.536 |  | 0.178 |  | -0.243 |  | 0.563 |  | 0.396 |  | 0.377 |

**Supplementary Table 6 - Spearman’s correlations between discrimination index and power spectra at different frequency bands in HD condition during exploration of the displaced object.** The second line shows each frequency band, the first column shows hippocampal areas (L: Left and R: Right). Subsequent columns show statistical results using Searman’s correlations test for correlation between discrimination index and band power at different frequency bands: the rho-value, p-value, for for each frequency band. The statistical spearman rho, and the p-values are shown for each area and frequency band, significant p-values are in bold with an asterisk.

|  |  | **Frequency band** | | | | | | | | | | |
| --- | --- | --- | --- | --- | --- | --- | --- | --- | --- | --- | --- | --- |
|  |  | **Theta** | | |  | **Slow Gamma** | | |  | **Fast Gamma** | | |
| **Area** |  | **rho-value** |  | **p-value** |  | **rho-value** |  | **p-value** |  | **rho-value** |  | **p-value** |
| **L CA1** |  | 0.700 |  | 0.233 |  | 0.500 |  | 0.450 |  | 0.500 |  | 0.45 |
| **R CA1** |  | -0.100 |  | 0.95 |  | 1 |  | **0.016*** |  | 0.900 |  | 0.083 |
| **L CA3** |  | -0.300 |  | 0.683 |  | 0.800 |  | 0.133 |  | 0.800 |  | 0.133 |
| **R CA3** |  | -0.300 |  | 0.683 |  | 0.300 |  | 0.683 |  | 0.400 |  | 0.516 |
| **L DG** |  | 0 |  | 1 |  | 0.100 |  | 0.950 |  | 0.100 |  | 0.95 |
| **R DG** |  | -0.900 |  | **0.009*** |  | 0.100 |  | 0.950 |  | -0.300 |  | 0.683 |

**Supplementary Table 7 - Spearman’s correlations between discrimination index and power spectra at different frequency bands in HD test during exploration of the stationary object.** The second line shows each frequency band, the first column shows hippocampal areas (L: Left and R: Right). Subsequent columns show statistical results using Searman’s correlations test for correlation between discrimination index and band power at different frequency bands: the rho-value, p-value, for for each frequency band. The statistical spearman rho, and the p-values are shown for each area and frequency band, significant p-values are in bold with an asterisk.
